# Metal arc welding and the risk of skin cancer

**DOI:** 10.1007/s00420-017-1248-5

**Published:** 2017-08-01

**Authors:** K. N. Heltoft, R. M. Slagor, T. Agner, J. P. Bonde

**Affiliations:** 1 Department of Occupational and Environmental Medicine, Bispebjerg Hospital, University of Copenhagen, Copenhagen, Denmark; 2 Department of Dermatology, Bispebjerg Hospital, University of Copenhagen, Copenhagen, Denmark

**Keywords:** Welder, Welding, Occupational, Skin cancer, Basal cell carcinoma, Squamous cell carcinoma, Melanoma, Ultraviolet radiation

## Abstract

**Objectives:**

Arc welding produces the full spectrum of ultraviolet radiation and may be a contributory cause of skin cancer; however, there has been little research into this occupational hazard. The aim of this study is to explore if metal arc welding increases the risk of malignant melanoma and/or basal cell carcinoma (BCC) and squamous cell carcinoma (SCC) on skin areas which may possibly be exposed (neck, head, and upper extremities).

**Method:**

A Danish national company-based historic cohort of 4333 male metal arc welders was followed from 1987 through 2012 to identify the risk of skin cancer. An external reference group was established including all Danish skilled and unskilled male workers with similar age distribution. Occupational histories were gathered by questionnaires in 1986 and information about skin cancer diagnoses [BCC, SCC, cutaneous malignant melanoma (CMM), and precancerous conditions, actinic keratosis (AK)] were gathered from the Danish Cancer Registry supplemented by the data from the Danish Pathology Register. Hazard ratios (HRs) were calculated in the follow-up period from 1987 until 2012 using Cox regression analysis and adjusted for baseline data regarding age and social group.

**Results:**

The adjusted HR and 95% confidence interval (CI) for skin cancer (all types) were 0.99 (CI 0.94–1.04) for welders. The adjusted HR for AK and BCC located only at neck was 2.49 (CI 1.03–5.99) for welders exposed >20 years (*n* = 5) and 2.46 (CI 1.02–5.94), respectively, for welders exposed >30 years (*n* = 5). No statistically significant difference was observed for SCC. The risk of CMM at the neck was also significantly elevated after 30 years of welding, but this is based upon only one exposed case.

**Conclusion:**

This study indicates that long-term exposure to metal arc welding may be related to increased risk of BCC and AK located exclusively at the neck. The study provides no support for the hypothesis that welding exposure increases the risk for skin cancer at other locations.

## Introduction

Exposure to outdoor ultraviolet radiation (UVR) from the sun is a well-known risk factor in the development of skin cancer (Armstrong and Kricker 2001; Wong et al. 2003). Although solar UVR is accepted as the major etiological factor, it is possible that non-solar sources of UVR (such as arc welding) may also be a contributory cause of malignancy. Estimates suggest that more than 3 million workers worldwide use welding in their work, and welding is still a major source of occupational UVR exposure (Baxter et al. 2010).

Arc welding produces the full spectrum of UVR including UVA (400–315 nm), UVB (315–280 nm), and UVC (280–100 nm). UVA penetrates the skin more deeply than UVB or UVC, but is less associated with DNA damage (Horneck 2000). However, epidemiological data indicate that UVA may be an important factor for the development of malignant melanoma (Moan et al. 2008). UVB is responsible for erythema and most of the DNA damage within skin cells, and consequently most of the resultant skin cancers (Gallagher and Lee 2006). Humans are rarely exposed to UVC, as solar UVC is readily absorbed in the atmosphere before it reaches the earth’s surface. In contrast, welders could also be exposed to UVC, as the short distance between the arc and the welder’s skin may not be sufficient to absorb most of the UVC (Baxter 2010; Dixon and Dixon 2004). Little is known of the damage that can be caused by UVC, but it may be as carcinogenic to skin as UVB (Merryman 1999).

The incidence of the three major types of skin cancer [basal cell carcinoma (BCC); squamous cell carcinomas (SCC); and cutaneous malignant melanoma (CMM)] has been increasing steadily over the past years. An association between cumulative UVR exposure and SCC has clearly been documented (Rosso et al. 1996); the association with BCC is partly the same, although sunburn is also known to be a risk factor (Gallagher and Lee 2006). CMM is believed to be correlated with sunburn and intermittent UVR exposure rather than the cumulative lifetime exposure (Armstrong and Kricker 2001; Agner et al. 2013). SCC, and sometimes also BCC, may be related to cumulative UVR during working hours, however, this is not the case for CMM, for which the pigmentation following cumulative UVR may even be a protective factor.

Welders are exposed to radiation both directly and indirectly (Tenkate and Collins 1997). In the welding environment, personnel are protected from UV exposure through the use of safety spectacles, welding hats, helmets, and industrial clothing. However, studies have shown that welding radiation might penetrate the welding gear from the side and bystanders can be exposed (Tenkate and Collins 1997). Furthermore, it has been suggested that UVR exposure inside welding helmets and on unprotected skin surfaces of welders exceed maximum permissible exposure (MPE) limit for UV radiation (Tenkate and Collins 1997). It is possible that welders are at greater risk of developing skin cancer than the general population, but there is a dearth of well-designed studies addressing this issue.

The suspicion linking welding with skin cancer originates from reports. Several case reports have been published of BCC and SCC occurring after years of arc welding (Currie and Monk 2000; Dixon 2007; Donoghue and Sinclair 1999; Kristensen and Sorensen 2015; Wolfe et al. 2013). Currie et al. reported on five cases of welders with non-melanoma skin cancer (NMSC) whose mean age at presentation was 52 years (Currie and Monk 2000). Another casuistic study reported a woman who had presented with numerous SCC on her hands and forearms after assisting her son with welding business (Dixon 2007). Three other case reports depicted welders developing SCC and BCC after unprotected arc welding (Donoghue and Sinclair 1999; Kristensen and Sorensen 2015; Wolfe et al. 2013). Although casuistic studies have demonstrated a potential risk of NMSC due to welding, the epidemiological evidence for an association between long-term arc welding and skin cancer is limited.

Three case–control studies of the risk of skin cancer from arc welding have been published. Emmett et al. observed that welders often had skin erythema and small scars from burns, but did not find increased incidence of skin cancer in welders (Emmett et al. 1981). As has been remarked, the welders examined were all well protected and young so the length of their exposure was relatively short compared to senior career workers (Dixon and Dixon 2004). It is conceivable that the UVR skin damage had not yet manifested as malignancy. Likewise, a case–control study assessed the potential association between non-solar UVR and the subsequent risk of BCC and SCC (Bajdik et al. 1996). They identified 406 patients with BCC and SCC (of which 118 had welded) and 406 age-matched controls (consisting of 107 welders). The study found no association between welding exposure and BCC and SCC. However, as the author remarked, the limited numbers of exposed cases and controls in the study restricted its statistical power (Bajdik et al. 1996). The third case–control study included 200 non-welders and 200 welders (Zamanian et al. 2015). They found a significant difference in the prevalence of actinic keratosis, but did not find a link between welding and the development of CMM. Unfortunately, the study had severe limitations due to the mean age of participants studied being only 36 years.

Large, well-designed studies are needed to clarify the uncertainty about the risk of skin cancer from arc welding. In addition, it is important to include long-serving career workers, who are likely to have accumulated greater welding UVR exposure.

The aim of this study is to investigate if metal arc welding increases the risk of CMM, BCC, SCC, and actinic keratosis (AK). It should be noted, however, that skin cancer is not considered to be the only potential adverse health effect of UVR exposure from welding. IARC has identified ocular melanoma in welders as a major health issue. The focus of this article is, however, only skin cancer. The present study will hence, to our knowledge, be the first large-scale 25-year follow-up study to assess the potential association between welding and the subsequent risk of skin cancer.

Considering the fact that malignant melanoma and maybe also BCC appear to be strongly related to intermittent UV radiation, one would expect a potential link between welding and especially BCC and malignant melanoma since welding causes high level of intermittent UVR radiation. However, welding also adds to the cumulative amount of UVR, which may increase the risk of SCC and AK.

Furthermore, studies indicate that welders are at greater risk of UV exposure (exceeding the MPE) mainly at skin areas not protected by industrial clothing (neck and upper extremities) (Tenkate and Collins 1997; Zamanian et al. 2015). A potential association would, therefore, be expected primarily at these locations.

## Methods

### Study population

The participants were selected from a cohort of 10,059 male production workers employed at Danish stainless steel or mild steel manufacturing companies for at least 1 year within the period of April 1964 to December 1984. The cohort was originally created with the aim of studying lung cancer risk among welders. Later, the cohort was used for studying infertility, asthma, cardiovascular diseases, and cataract (Bonde et al. 1990; Hansen et al. 1996; Ibfelt et al. 2010; Kristiansen et al. 2015; Slagor et al. 2016; Sorensen et al. 2007). A more detailed description of the metal welders’ cohort is described elsewhere (Hansen et al. 1996; Ibfelt et al. 2010; Slagor et al. 2016; Sorensen et al. 2007).

The cohort was established in 1984 based upon company roster and records from the Danish Pension Fund (Hansen et al. 1996). In Denmark, all employees are obligatory members of the Danish Pension Fund, which made it possible to identify former and present employees using specific company codes. The cohort members were enrolled at 75 companies in Denmark employing about 60% of all Danish stainless steel (SS) welders and at five large mild steel (MS) companies. Due to possible asbestos exposure, shipyards were excluded. The participants were employed as MS welders, SS welders, SS grinders or non-welding/grinding production workers. In autumn 1986, all the workers were sent questionnaires on lifetime occupational exposures, smoking, and drinking habits. The questionnaires were eliciting information on work history in the years 1950–1985, years of welding per decade in MS and/or SS, and hours of welding per day. The information about welding processes was checked by company visits and roster of employees when the cohort was created.

The questionnaire also provided information on used welding methods [manual metal arc (MMA), metal active gas (MAG), tungsten inert gas (TIG) and others]. TIG and MAG welders are, in general, more exposed to UVR, since these processes produce less welding fumes than MMA. However, most metal workers use more than one method during work; therefore, it was not possible to investigate the separate effects of TIG, MAG, and MMA and the subsequent risk of skin cancer. Unfortunately, there was no information about the UV spectrum to which the welders were exposed during welding nor information about the use of welding gear.

**Fig. 1:**
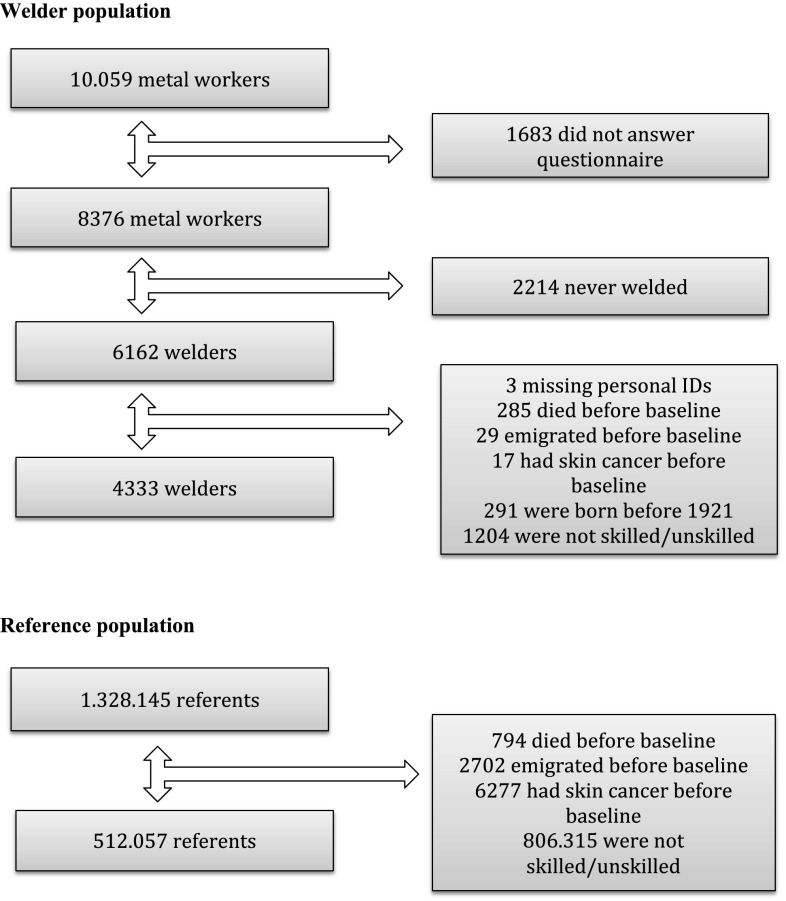
Flow diagram welders and referents welder population

Baseline was defined as January 1, 1987. In total, 8376 workers (83%) filled in the questionnaire. A total number of 4043 persons were excluded (2214 never welded, 3 missing personal IDs, 285 died before baseline, 17 had skin cancer before baseline, 291 were born before 1921, and 1204 were not skilled/unskilled. This left 4333 metal arc welders for final analysis (Fig. 1).

Social group was identified from Statistics Denmark’s registration in 1985 and approximately 80% of the welders belonged to social group, skilled and unskilled. The categories for social groups were chief executive, self-employed, white-collar worker, skilled blue-collar worker, unskilled blue-collar worker, student or retired. All other social groups were excluded from both welders and referents to secure a better match between welders and reference group.

An external reference group was established through the Danish population registry including all males born between January 1, 1921 and April 1, 1964. The reference population included males who had worked for at least 1 year in any Danish company between April 1964 and December 1984, and who were alive on January 1, 1985, resulting in a total of 512,057 referents (Fig. 1).

### Outcome

The outcome was BCC, SCC, CMM, and precancerous conditions (actinic keratosis), which was identified through the Danish Cancer Registry supplemented by the data from the Danish Pathology Register. The Danish Cancer Registry is nationwide and includes virtually complete data of all incident cancers in Denmark since 1943, including details on morphology and histology (Gjerstorff 2011; Jensen et al. 1985). Tumors are coded according to a Danish version of the 10th revision of the International Classification of Diseases (ICD-10), and in addition, before 1978, according to the 7th revision of the ICD.

The Danish Pathology Register contains detailed records of all pathology specimens analyzed in Denmark since 1997, as well as records of specimens from some pathology departments dating back to the 1970s. Date of request and diagnoses based on the Danish SNOMED codes are reported. The SNOMED codes include information on anatomical location and histology. The linkage was performed using the personal identification number.

Welders’ and referents’ personal identification numbers were linked with Statistics Denmark registers with birth, emigration, death, and social group.

### Statistical analysis

The risk of diagnosis for melanoma and non-melanoma skin cancers (NMSC) in welders and the reference group was examined in the period January 1, 1987 through December 31, 2012.

To account for potential differences in follow-up time in exposed and unexposed, we used survival analyses (Cox regression) to estimate hazard ratios (HRs) with 95% confidence intervals (CIs) for CMM, NMSC, and AK according to welding (yes/no) and cumulative duration of welding. The outcome date was taken as the date of the first registration of skin cancer diagnosis during follow-up. The lifelong exposure to welding was categorized into: 1–10 years, 11–20 years, 21–30 years, and 31–48 years as the independent variable and all analyses were adjusted for potential confounders recorded in 1986: age and social group (skilled/unskilled). Data were censored by date of death, first date of emigration or diagnosis of skin cancer before baseline, which ever occurred first. Due to the existence of the personal identification number, we were able to do a follow-up study with no loss to follow-up.

We tested the proportional hazard (PH) assumption for the Cox regression by inspection of plots of the survival distribution function against follow-up time and found no indication of violation of this assumption (no crossing of the survival curves). Furthermore, the PH assumption was numerically tested for interaction between exposure and time prior to analysis. The result was not statistically significant (*p* = 0.1041) implying that welding has a constant impact on the hazard over time. The data analysis was carried out using SAS 9.3 software (proc PHREG and proc LIFETEST).

## Results

In total, 4333 male welders were included for final analysis. The mean age at entry to follow-up was 40.64 years among exposed welders and 39.67 years among referents. 223 (5.15%) cases of skin cancer were identified among welders and 24,225 (4.73%) cases among referents. Table 1 describes the selected descriptive characteristics of the welders and referents.

**Table 1:** Characteristics of welders and referents when the cohort was established in 1987

	Welders	Referents
At baseline, *N*	4333	512,057
Mean years of follow-up, years (SD)	22.85 (6.34)	22.61 (6.42)
Mean age at baseline, years (SD)	40.64 (10.17)	39.67 (11.45)
Mean age onset of skin cancer, years (SD)	63.00 (10.30)	63.23 (11.03)
Age <25, *N* (%)	228 (5.26)	46,200 (9.02)
Age 26–34, *N* (%)	1138 (26.26)	160 826 (31.41)
Age 35–44, *N* (%)	1589 (36.67)	143,083 (27.94)
Age 46–54, *N* (%)	928 (21.42)	94,791 (18.51)
Age 56–66, *N* (%)	450 (10.39)	67,157 (13.12)
Dead, *N* (%)	1052 (24.28)	138,848 (27.12)
Immigrated, *N* (%)	122 (2.82)	14,734 (2.88)
Skilled, *N* (%)	3310 (76.39)	245,292 (47.90)
Unskilled, *N* (%)	1023 (23.61)	266,765 (52.10)
Diagnosis skin cancer (total number of patients), *N* (%)	223 (5.15)	24,225 (4.73)
Diagnosis skin cancer (all cases)	4434	35,285
Diagnosis of basal cell carcinoma, *N* (%)	177 (4.08)	17,980 (3.51)
Diagnosis of squamous cell carcinoma, *N* (%)	37 (0.85)	4691 (0.92)
Diagnosis of non-melanoma skin cancer, *N* (%)	200 (4.62)	21,357 (4.17)
Diagnosis of malignant melanoma, *N* (%)	32 (0.74)	3475 (0.68)
Diagnosis of actinic keratosis, *N* (%)	76 (1.75)	8934 (1.74)
Welded 1–10 years, *N* (%)	1725 (39.81)	–
Welded 11–20 years, *N* (%)	1373 (31.69)	–
Welded 21–30 years, *N* (%)	886 (20.45)	–
Welded 31–48 years, *N* (%)	349 (8.05)	–
Total number of years welding, years	64,894	–

The mean age at the onset of skin cancer among the 4333 welders was 63.00 years, similar to the mean age of 63.23 years among the referents.

We found no significant association between total years of welding and risk of skin cancer (all types), when adjusted for age and social class (HR 0.99 (95% CI 0.94–1.04)). In addition, no statistically significant differences were observed when the outcome was branched in BCC, SCC, CMM, and AK and the welding exposure was stratified into the groups 1–10, 11–20, 21–30, and 31–48 years, respectively (Table 2).

**Table 2:** HRR’s and 95% CIs for skin cancer among 4333 welders according to welding exposure

	Welding exposure	Person–years	*N*	HR crude	CI 95%	HR adjusted^a^	CI 95%
Skin cancer (total)	Reference	11,790,836	35,285	1.00	–	1.00	–
	1–10 years	40,397	98	0.79	0.65–0.97	1.02	0.84–1.25
	11–20 years	32,903	83	0.83	0.67–1.03	0.84	0.67–1.04
	21–30 years	20,751	92	1.48	1.21–1.82	1.00	0.82–1.23
	31–48 years	6890	51	2.74	2.08–3.61	1.00	0.76–1.31
Basal cell carcinoma (total)	Reference	11,895,651	17,980	1.00	–	1.00	–
	1–10 years	40,640	59	0.94	0.73–1.22	1.19	0.92–1.54
	11–20 years	33,143	42	0.82	0.61–1.12	0.82	0.61–1.11
	21–30 years	20,981	49	1.56	1.18–2.06	1.06	0.80–1.41
	31–48 years	7071	27	2.74	1.88–3.99	1.05	0.72–1.54
Squamous cell carcinoma (total)	Reference	12,001,922	4691	1.00	–	1.00	–
	1–10 years	41,153	9	0.55	0.28–1.05	0.81	0.42–1.56
	11–20 years	33,390	10	0.75	0.41–1.40	0.87	0.47–1.62
	21–30 years	21,263	11	1.33	0.74–2.40	1.01	0.56–1.82
	31–48 years	7320	7	2.65	1.26–5.56	0.95	0.45–1.98
Non-melanoma skin cancer (total)	Reference	11,868,657	21,357	1.00	–	1.00	–
	1–10 years	40,586	68	0.85	0.67–1.08	1.12	0.88–1.42
	11–20 years	33,091	53	0.82	0.62–1.07	0.84	0.64–1.09
	21–30 years	20,928	61	1.53	1.19–1.96	1.05	0.82–1.35
	31–48 years	7030	34	2.73	1.95–3.82	1.02	0.73–1.43
Malignant melanoma (total)	Reference	12,004,146	3475	1.00	–	1.00	–
	1–10 years	41,148	8	0.66	0.33–1.32	0.73	0.36–1.45
	11–20 years	33,346	13	1.33	0.77–2.29	1.25	0.72–2.15
	21–30 years	21,289	5	0.81	0.34–1.95	0.60	0.25–1.45
	31–48 years	7311	6	3.02	1.36–6.74	1.59	0.72–3.55
Actinic keratosis (total)	Reference	11,973,308	8934	1.00	–	1.00	–
	1–10 years	41,077	22	0.20	0.46–1.07	0.92	0.61–1.40
	11–20 years	33,348	17	0.67	0.42–1.08	0.69	0.43–1.11
	21–30 years	21,159	26	1.60	1.08–2.37	1.08	0.73–1.59
	31–48 years	7273	11	2.27	1.26–4.09	0.72	0.42–1.38

^a^ Adjusted for age and social class

We investigated the separate effects of welding exposure on particularly exposed skin areas (neck, face, head, and upper extremities). Table 3 shows HR analyses performed among the subgroups of skin cancer diagnoses located at the neck. A significantly higher risk for BCC was found for welders exposed to welding more than 30 years (HR 2.46; 95% CI 1.02–5.94). Likewise, the incidence of AK diagnoses located at the neck was higher among welders exposed for more than 20 years (HR 2.49; 95% CI 1.03–5.99). Regarding CMM located at the neck, only one exposed case makes it difficult to arrive at any conclusions.

**Table 3:** HRR’s and 95% CIs for skin cancer locations at neck among welders according to welding exposure

	Welding exposure	*N* (%)	HR crude	CI 95%	HR adjusted^a^	CI 95%
Basal cell carcinoma (location neck)	Reference	1281	1.00	–	1.00	–
	1–10 years	7	1.58	0.75–3.32	2.06	0.98–4.33
	11–20 years	4	1.11	0.42–2.97	1.11	0.42–2.98
	21–30 years	3	1.33	0.43–4.13	0.89	0.29–2.78
	31–48 years	5	6.91	2.87–16.62	2.46	1.02–5.94
Squamous cell carcinoma (location neck)	Reference	244	1.00	–	1.00	–
	1–10 years	0	–	–	–	–
	11–20 years	2	2.88	0.72–11.58	3.36	0.84–13.51
	21–30 years	1	2.33	0.33–16.54	1.58	0.22–11.29
	31–48 years	0	–	–	–	–
Malignant melanoma (location neck)	Reference	29	1.00	–	1.00	–
	1–10 years	0	–	–	–	–
	11–20 years	0	–	–	–	–
	21–30 years	0	–	–	–	–
	31–48 years	1	60.69	8.28–444.82	18.55	2.44–141.12
Actinic keratosis (location neck)	Reference	732	1.00	–	1.00	–
	1–10 years	2	0.79	0.20–3.15	1.04	0.26–4.15
	11–20 years	2	0.97	0.24–3.89	0.98	0.25–3.94
	21–30 years	5	3.91	1.62–9.43	2.49	1.03–5.99
	31–48 years	0	–	–	–	**–**

^a^ Adjusted for age and social class

The results did not show any evidence of significantly elevated risk of skin cancer located at face, head, and upper extremities (data not shown).

A sensitivity analysis only including welders and referents older than 50 years was performed. We found no significant association between total years of welding and risk for skin cancer. But as described above, a significantly higher risk for BCC and AK located at the neck was found for welders.

## Discussion

In this follow-up study of male welders, we assessed the association between welding exposure and the subsequent risk of BCC, SCC, CMM, and AK. When skin cancer diagnoses were stratified according to location, a significant difference in the incidence of BCC and AK located at the neck was found in relation to long-term arc welding. It should be noted; however, that no clear dose–response relationship appeared (Table 3). Furthermore, the limited number of cases in these subgroups (based on location) restricts the statistical power. Consequently, the elevated risk might be incidental. Nonetheless, the results indicate that the skin areas not fully protected by industrial clothing and welding helmet (such as the neck) may be particularly exposed to UV radiation. It is plausible that the neck during welding carries a higher risk of developing malignancy.

It is well known that skin cancer occurs especially on sun-exposed areas of the body (neck, head, forearms, and hands); however, these results suggest that welders receive higher UV exposure at the neck compared to the referents leaving this profession at greater risk.

Unfortunately, due to lack of information, we were not able to categorize welders as outdoor or indoor workers. Nonetheless, the welders admitted into this cohort must be considered as primarily indoor workers, as the participants were employed at machine shops and welders at shipyards were excluded. The fact that we found an increased incidence of tumors on the neck and not in the face, a localization where outdoor workers often develop skin cancers, indicates that radiation from welding causes the tumors rather than sun exposure due to outdoor work.

Regarding other locations, we did not find increased incidence of skin cancer cases according to welding exposure.

The main strengths of the present study are the size of the cohort of welders and reference group, and the relatively large number of skin cancer cases. In addition, due to the existence of the personal identification number, which is used in all health registries and secures correct linkages between registries, we were able to do a follow-up study with virtually no loss to follow-up. Another advantage is the long follow-up period up to 25 years.

NMSC data are not routinely collected by cancer registries, but in Denmark, we have an extensive registration of NMSC in two nationwide population-based registries, and the completeness of NMSC registration is presumed to be high by including multiple settings in the health care system (Gjerstorff 2011). Furthermore, all residents in Denmark have equal, free admission to hospital and general practitioners.

Still, this study has limitations. We were not able to adjust for skin type, eye color, and number of freckles, which are well-established risk predictors for skin cancer (Gallagher and Lee 2006; Diepgen and Mahler 2002). However, it seems unlikely that the distribution of these risk predictors should differ between welders and referents. In addition, these individual risk factors are more important for CMM.

An additional limitation is that we lacked information on welders and outdoor workers in the reference group. This could lead to non-differential misclassification of exposure and a consequent underestimation of risk. Welders constitute less than 5% of the entire population of male skilled and unskilled workers in Denmark (Beskæftigelses ministeriet 2013; Rosendahls-shulz Grafisk and Miljø- og socialsekretariatet 2010). As a result, exposure misclassification due to presence of welders in the reference population is considered of minor importance.

As regards occupational skin cancer in outdoor workers, a review (addressing occupational skin cancer) identified six studies from latitudes similar to Denmark (Agner et al. 2013). Three out of six studies found a positive association between occupational solar UV exposure and incidence of BCC, although the risk increase was statistically significant only in one study (129 CI % 113–142) (Agner et al. 2013). Regarding SCC, a total of five studies were identified, of which four found a positive link between occupational solar UVR and incidence of SCC. In relation to CMM, seven studies were identified, of which two found a positive association. However, risk was significantly elevated only in one study (Agner et al. 2013). Consequently, the referents in the present study may not truly be unexposed and this bias could induce underestimation.

Data on occupational exposure had been collected prior to the planning of this study, and diagnoses of BCC, SCC, and malignant melanoma were obtained independently from the Danish Cancer Registry and the Danish Pathology Register. The prospectively obtained information about welding processes is reliable because exposure status was checked by company visits and rosters of employees when the cohort was created in 1985 (Hansen et al. 1996). All included welders have welded to some extent. However, the welding exposure after 1985 is not estimated in this study. The lack of information on welding after 1985 consequently leads to a possible misclassification of exposure, which may bias the risk estimate toward null. Consequently, we performed a sensitivity analysis only including welders and referents older than 50 years at baseline. The sensitivity analysis showed similar results, indicating that this possible misclassification of exposure is of minor importance.

Higher socioeconomic status has been associated with an increased incidence of BCC and CMM (Idorn et al. 2013; Lear et al. 1997; van der Aa et al. 2011). Social class is associated with highly paid occupations and as a result more frequent overseas travels with holidays in the sun. Furthermore, higher socioeconomic status is associated with increased visits to physicians consequently leading to a better registration of BCC and CMM (Agner et al. 2013). In the present study, the reference group had a higher percentage of unskilled workers; however, the analyses were adjusted for social group, and confounding by social class seems unlikely.

Another potential confounder is sun exposure during leisure activities. We do not have information on sunbathing, outdoor activities, use of sunscreen, etc. Nonetheless, it seems unlikely that welders would be exposed more or less than the reference group with a similar socioeconomic position.

In general, BCC and SCC are being studied much more rarely than melanoma, because of no or incomplete registration of NMSC in various cancer registries (Lucke et al. 1997). Hence, to our knowledge, the present study is the first large-scale follow-up study to assess the potential association between welding and the subsequent risk of NMSC and malignant melanoma.

## Conclusions

In this relatively large Danish cohort study with 25 years of systematic follow-up, we found increased incidence of BCC and AK located exclusively at the neck according to long-term arc welding. This finding is plausibly explained by the higher UV exposure at the neck, since this location may not be sufficiently protected by welding helmet and clothing. Consequently, the results indicate that especially the neck during welding operations requires further protection.

This may be achieved through the use of sunscreen and more appropriate industrial clothing.

## Summary

The purpose was to explore if UVR exposure from metal arc welding increases the risk of malignant melanoma, basal cell carcinoma, squamous cell carcinoma, and/or actinic keratosis. The present study of 4333 welders and 512,057 referents indicates that long-term exposure to arc welding is related to increased risk of basal cell carcinoma and actinic keratosis located at the neck. The study provides no support for the hypothesis that welding exposure increases the risk for skin cancer at other locations. Data were adjusted for age and social group.
